# CBOs’ Capacity and Resilience on Serving Older AA and NHPI Adults During the Pandemic

**DOI:** 10.1093/geroni/igab046.3361

**Published:** 2021-12-17

**Authors:** Eun Jeong Lee, KeliAnne Hara-Hubbard, Sonia Bishop, Jae Lim, Linda Ko

**Affiliations:** 1 Asian American Resource and Information Network, Wood Ridge, New Jersey, United States; 2 University of Washington, Seattle, Washington, United States; 3 Fred Hutchinson Cancer Research Center, Seattle, Washington, United States; 4 Swedish Health Services, Seattle, Washington, United States

## Abstract

Community-based organizations (CBOs) are essential settings for older Asian American (AA) and Native Hawaiian Pacific Islander (NHPI) adults for accessing culturally and linguistically appropriate services and connecting with and support each other. This study examined the impact of the COVID-19 pandemic on CBOs’ ability to serve older AA & NHPI adults. This mixed methods study (survey and semi-structured interviews) used a sequential exploratory design. We recruited 65 leaders and staff members from 40 CBOs serving older AA & NHPI adults nationally. Descriptive analysis was conducted with the survey data followed by thematic analysis of the interview data. Many CBOs were impacted by the increased demands for services (80%) and created new services (75%) while experiencing programming disruption (69%), decreased staffing (55%), and loss of revenue (38%). Some CBOs temporarily closed their organizations (38%), while others closed permanently (3%). To remain in operation, many CBOs (65%) increased their online presence, hired staff (52%), and recurred to financial reserves (20%). The semi-structured interviews identified four themes: 1) CBOs resourcefulness to acquire and share resources, 2) technology as a connector for CBOs and an isolator for older adults, 3) heightened racial discrimination against Asians, and 4) emergence of multi-level resilience (personal/ community/organizational). CBOs experienced disruption in their operation, and heightened racial discrimination during the pandemic. Yet, CBO’s ability to remain resilient was critical to continue to provide key programs for older adults. Future studies may want to examine evolving needs of CBOs as they adjust to new public health challenges during the pandemic.

